# New products

**DOI:** 10.1155/S1463924601000153

**Published:** 2001


Journal of Automated Methods & Management in Chemistry, Vol. 23, No. 4 (July–August 2001) pp. 129–132

### New products

### Twister™ swiftly becoming the sample storage industry standard

Zymark Corporation announces the Twister Universal Microplate Handler, a low-cost, bench-top, microplate handler designed to standardize laboratory applications for microplate readers, washers and liquid handlers. Twister individually moves up to 20 microplates from an input rack to the plate locator on the instrument. An optional loader accommodates up to 60 additional plates. Twister software initiates the scientific instrument's method. After completion, Twister retrieves the plate from the instrument and places it into an output rack. The software can cycle plates in predetermined intervals to accommodate kinetic applications. Twister can also be interfaced with a wide variety of devices from the industry's leading instrumentation companies, and thus equips scientists with small bench-top workstations. It also provides an easy-to-use means of unattended operation.
Companies, such as Cartesian Technologies, Inc., have chosen to partner Twister with their existing automation, the ProSys™ 5510 Production Microarraying System. The ProSys 5510 is a high capacity DNA microarraying workstation used for creating high-density arrays in functional genomics research. The ProSys system is configured with a pin array transfer head, 100-slide nest and up to 48 micro spotting pins all enclosed in a humidity-controlled environment. Its remarkably precise high-density print heads are capable of printing up to 82 000 spots on a single slide from 96 to 384 well source plates.

*For more information contact: Tom Maltais, Zymark Corporation, Hopkinton, MA, USA: Tel: + 1 508 497 6541; e-mail: Tom.Maltais@zymark.com*

### EDS and INCA

Exceptionally accurate energy dispersive spectrometry (EDS) analysis is now guaranteed with the latest version of the INCA Microanalysis Suite from Oxford Instruments. Excellent EDS performance is now a reality, for example when using low-beam energy or where X-ray peaks are overlapped.

The quantitative EDS analysis by the INCA Energy system is now enhanced by the introduction of Profile optimization, an algorithm for improving standard profiles for best peak deconvolution. X-ray peaks for low-energy L and M lines can be automatically identified and labelled using a new X-ray line database based on the latest research.

This newest release of the Microanalysis Suite, which offers EDS, wavelength dispersive spectrometry (WDS), and electron backscatter diffraction (EBSD), both singly or in combination, in an easy-to-use format via a single PC, now includes 'INCA SiteLock' and INCA Export'.

INCA SiteLock (which is available for INCA Energy & INCA Energy TEM) is an operator-independent program that, once initiated, automatically performs beam drift correction, producing sharper images even on long data runs. Maps, linescans and spectra are all improved, with drift correction available at both high and low contrast and high and low magnifications, as selected by the operator. In addition, information from INCA SiteLock is automatically saved with the captured data.

Users can also now export an EDS spectrum, image or map to a local or network folder to be viewed and manipulated by a user who does not have INCA installed. By selecting the INCA Export button from the data tree, the operator creates a file so small that it can easily be distributed via e-mail. Using the new INCA Viewer software—which can be downloaded free of charge from the Oxford Instruments website [www.oxford-instruments.com]— recipients can view and manipulate data on their own PC with the full functionality of INCA but without having to install INCA themselves.

A new Peak Label Editor also allows the user to select X-ray lines for labelling element peaks in a spectrum, providing clearer spectra and more flexible spectrum display, and a Check Total mechanism indicates whether peak and background content is consistent with the elements and indicates validity of the analysis when normalization has been used. Data export to Quartz PCI, a microscopy-oriented database that can be used for storage, sorting and retrieval of images and associated text files, will also be possible.

*For further information contact: Oxford Instruments Analytical, Halifax Road, High Wycombe HP12 3SE, UK. Tel: + 44 (0)1494 442255; Fax: + 44 (0)1494 524129*

### Electrode monitoring system for glass furnace with an interactive touch-screen display

An innovative monitoring system for the electrodes in glass production furnaces has been developed by F.I.C. (UK) Ltd of Penzance. F.I.C. is an international electroheat specialist with wide experience of supplying electric furnace systems to glass producers all over the world. The company's newly launched Electrode Maintenance Unit (EMU) continuously monitors and displays electrode performance, allowing wear or breakage to be picked up early enough to avoid refractory erosion or damage. The heart of the unit is based on Eurotherm's versatile Visual Supervisor control system, providing a high level of operator information, with touch-screen display.

The EMU allows glass production personnel to monitor fully and determine the state of wear of the electrodes at

*Journal of Automated Methods & Management in Chemistry* ISSN 1463–9246 print/ISSN 1464–5068 online © 2001 Taylor & Francis Ltd
http://www.tandf.co.uk/journals
DOI: 10.1080/14639240110092459

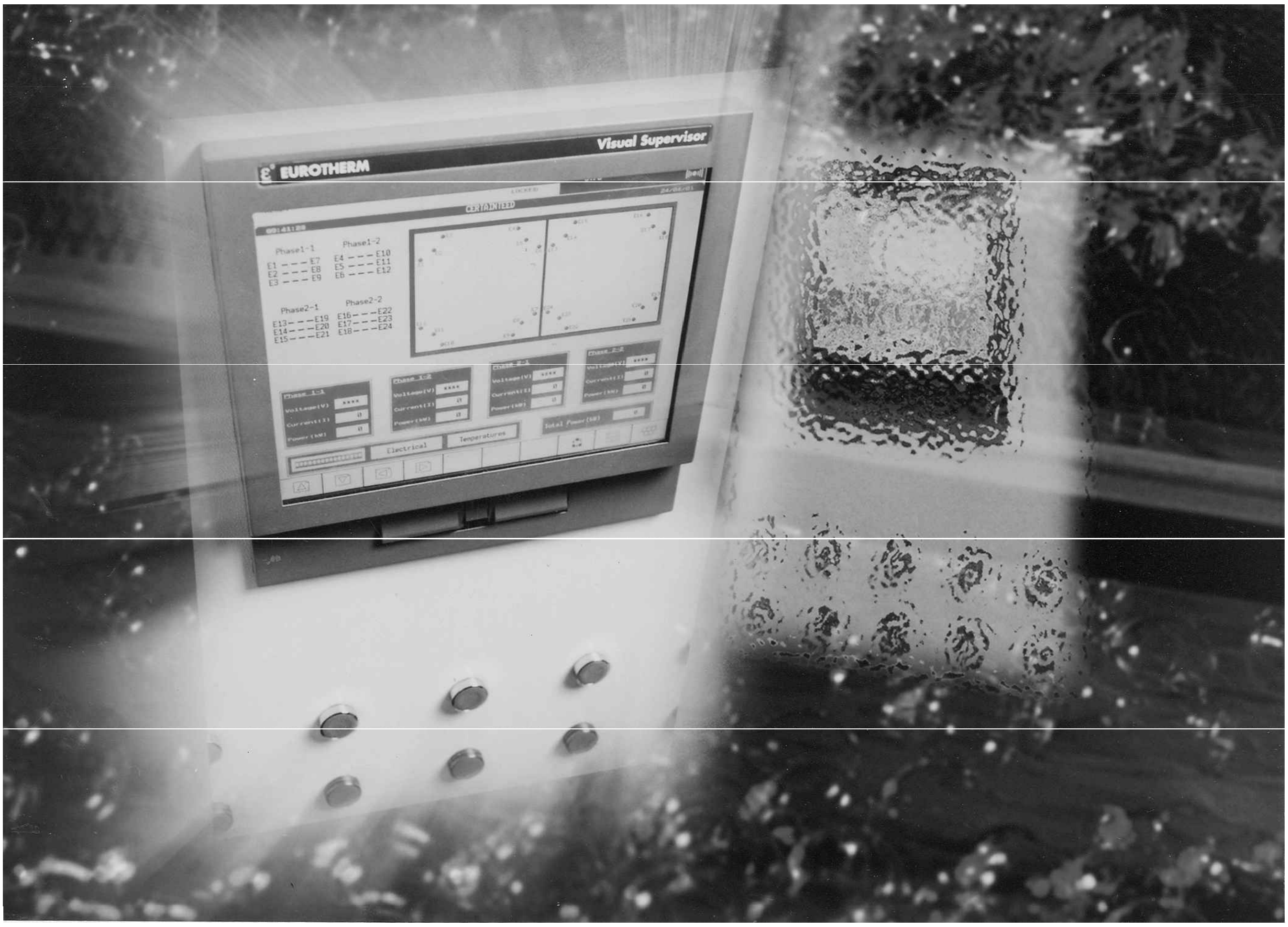

*An innovative monitoring system for the electrodes in glass production furnaces has been developed by F.I.C. (UK) Limited of Penzance. The company's newly launched Electrode Maintenance Unit (EMU) continuously monitors and displays electrode performance, allowing wear or breakage to be picked up early enough to avoid refractory erosion or damage. The heart of the units is based on Eurotherm's versatile Visual Supervisor control system, providing a high level of operator information, with touch-screen display.*

any time during the furnace campaign. Taking advantage of flash memory, it keeps a continuous record of electrode voltage and current, and is fully alarmed to alert operators of any problems detected with the electrodes. System versatility is such that electrode holder temperatures are monitored as well as furnace voltage and current parameters.

Both control and display functions within the EMU are provided by Visual Supervisor, a multifunction process controller, data logger, set-point programmer and interactive touch-screen display combined in one unit. The touch-screen technology allows rapid user access to each monitoring mode and user-friendly display software ensures both ease of use and clear system graphics. Visual Supervisor can perform both continuous and sequential control. It also features comprehensive alarm and event management, powerful trending, set-point programming and local data logging facilities.

Measurement of both electrode and furnace parameters is handled through an adjacent Eurotherm 2500 System, which provides high-performance, high-accuracy I/O routing from furnace thermocouples as well as electrode current and voltage transducers. The unit incorporates a number of unique hardware and software interactions that provide fast, accurate response times for analogue process control systems of the EMU type.

Undetected electrode wear or electrode breakage will lead to serious refractory erosion and possible glass leaks, thus shortening furnace life if not attended to promptly. Most electro-heat specialists offer breakage and wear detectors with varying degrees of reliability, but generally cannot determine the precise wear of multi-electrode poles accurately. This is because multiple electrodes connected to the same side of the transformer winding all detect the same signal.

Simple measurements such as determining the change in resistance are also unsatisfactory because electrode wear is not always uniform across the same circuit. Thus, precisely determining individual electrode wear on each side of the transformer winding has, until now, been for all practical purposes impossible.

Similarly, probes used to measure relative voltage across the system can only average out the effect of wear on the multi-electrode poles. This uncertainty can lead to over insertion of the electrode and the increased probability of breakage or, due to under insertion, increased current density close to the sidewall and, hence, accelerated wear of the refractory.

The F.I.C. EMU overcomes these problems, as specially derived algorithms ensure that all multi-electrode poles are balanced before a general overall push, which resets the electrodes to their datum levels. The software allows for breakage detection, balancing and wear calculation, thus ensuring total electrode supervision and maintenance.

F.I.C. offers a comprehensive service, commencing with modelling if required, and including design, manufacture and supply, plus on-site installation and commissioning. It has been an ISO9001 approved company since 1993 and was the first in the industry to receive accreditation.

Eurotherm Ltd is part of the Automation Division of Ivensys Plc, one of the world's leading automation and controls companies. Eurotherm is a major supplier to the world's processing and manufacturing industries.

*For further information contact: Kerrie Shields, Eurotherm Ltd, Faraday Close, Durrington, Worthing, West Sussex BN13 3PL, UK. Tel: + 44 (0)1903 268500; Fax: + 44 (0) 1903 695666; e-mail: info@eurotherm.co.uk; URL http://www.eurotherm.co.uk; or Steve King, F.I.C. (UK) Ltd, Merlin Works, Cuxhaven Way, Long Rock, Penzance, Cornwall TR20 8HX, UK. Tel: + 44 (0) 1736 366 962; Fax: + 44 (0) 1736 351 198; e-mail: general@fic-uk.com*

**E-beam control and large-scale orientation mapping with INCA Crystal**

Superb large-scale crystal orientation maps (COMs) obtained at speeds of up to 60 000 pixels per hour using fully corrected low-magnification mapping are now achievable from a new version of the INCA Crystal electron backscatter diffraction product from Oxford Instruments Analytical.

INCA Crystal, the latest release of the EBSD system, uses the INCA micro-analysis platform, which has benefits such as on-line help and a navigator system that leads less expert users in a clear and structured way through the portfolio of analysis options. An additional benefit is that all INCA Crystal systems can be extended to provide combined INCA energy dispersive (EDS) and wavelength dispersive (WDS) systems, or can be added later to existing INCA Energy/Wave systems. Switching between the three techniques is performed due to powerful, interactive and user-friendly software.

Until now, EBSD in the scanning electron microscope (SEM) has been regarded as an alternative *micro*-technique, compared with conventional X-ray diffraction—the established macro technique for texture and orientation studies. However, with the latest advance in INCA Crystal, EBSD becomes very much a complementary technique to conventional X-ray diffraction, with the facility to produce large-scale COMs. This provides scientists and engineers not only with direct measurements of local orientation— which can be correlated with material properties or failures—but also with large-scale orientation mapping for better comparison with X-ray diffraction data and statistically significant texture analysis.

For the first time, INCA Crystal allows users to perform fully corrected low magnification mapping for macro data collection *using beam control*. (The conventional approach is to use mechanical stage control, but this is slow and can be inaccurate, and often requires the use of expensive extra hardware.) INCA Crystal uses beam control to scan very large areas on the sample, together with dynamic focus, tilt and calibration compensation to overcome the attendant problems with large-area beam scanning. The result is large-scale COMs mapped at high speed with simple set-up—and without the need for expensive stage hardware.

Texture analysis has always been widely accepted as the domain of the expert crystallographer, where years of experience pay dividends in spotting texture trends, using complicated types of plot. The revolutionary approach of INCA Crystal is not only simple and quick, but also extremely effective at isolating different texture components.

*For more information contact: Oxford Instruments Analytical, Halifax Road, High Wycombe, HP12 3SE, UK. tel: + 44 (0)1494 442255; Fax: + 44 (0)1494 524129*

**3A approved pumping solutions for the dairy industry**

Growing concerns about food hygiene, together with increasingly stringent regulations and European standards, compel dairy manufacturers to observe scrupulous cleaning and sterilization routines to avoid cross-contamination. In the past, pump designs have not leant themselves to speedy stripdown and, particularly where there is a high mix of products, downtime for cleaning has represented a costly overhead. To help solve the problem, Flux Pumps's barrel and container pumps meet 3A and sanitary specifications with dramatically reduced downtime for cleaning. The 3A classification is there to raise hygiene levels in sectors such as dairy, food and pharmaceuticals processing. To meet the regulations, machinery parts that are in contact with the product must be polished to a very high finish. Flux includes versions meeting this requirement within its range of stainless steel progressive cavity (PC) and axial flow pumps and, having obtained 3A approval for its F 560S AAA and F427 models, Flux Pumps is now in a position to supply the dairy market.

In general terms, PC pumps are recommended for handling high-viscosity media—it is worth considering a PC pump for any product with viscosity > 600 mPa s. PC pumps are also preferred for handling fragile media, e.g. to avoid separation of emulsified products such as mayonnaise or damage to components such as fruit. Because the rotational speed is relatively low, very little energy is imparted to the medium, so that its integrity is protected. This is in contrast to a centrifugal pump that operates at relatively high speed and imparts high energy to the medium in an action resembling that of a liquidizer. Such a high level of mechanical stress can

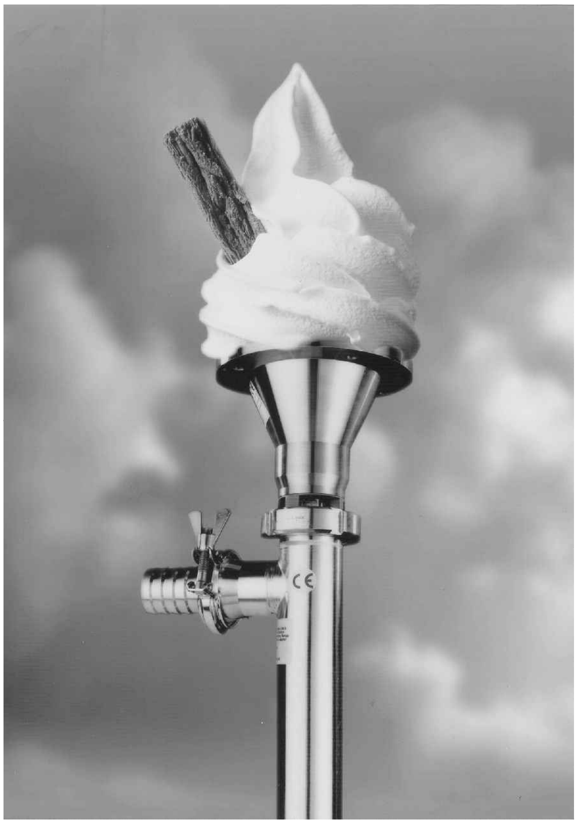

*Flux Pumps has invested in the development of pumping solutions for dairy applications that are unique in their class.*

result in physical degradation of fragile media. Products being handled by Flux PC pumps at a steady flow include many that have proved problematic in the past such as fruit purées, pie fillings, glucose, syrups, molasses, jams and mincemeat. When mounted vertically, the pumps have successfully overcome the problems associated with dispensing fondant into food production. Food-processing applications include vertical pumping of milk derivatives from IBCs into hoppers for introduction into food mixes. A further consideration with PC pumps is that their operation is independent of pressure, which means that when pumping liquids from barrels, the flowrate remains virtually the same, irrespective of liquid level.

Flux's PC pumps are an inexpensive and versatile addition to the alternatives available for food processing, for use with barrels, containers and other open-top process vessels. They are primarily, though not exclusively, designed for portable applications. Currently, 90% of Flux PC pumps are used in the food industry. Because many of the applications for this type of pump demand scrupulous hygiene, PC pumps must lend themselves to fast and easy stripdown for cleaning. The pumps are designed with minimum parts count and can be stripped down, cleaned and reassembled in 10–12 minutes. Also PC pumps provide a very economical way of carrying out operations which hitherto required a complicated, high-spec solution, often awkward to strip down and clean.

Flux Pumps' F 560S AAA progressive cavity pump handles viscosities up to 80 000 mPa s. For dairy use, however, the lightweight planetary gear version, handling viscosities up to 30 000 mPa s with flowrates up to 50 litres min$^{-1}$, will usually be adequate. Flux will work with customers to tailor the F 560S AAA to particular requirements.

The new version of Flux's F427 sanitary pump for barrel or container applications is ideal for processing a variety of liquids or colours, and quick-drying or film-forming liquids, up to a maximum operating temperature of 120°C. Wetted parts are manufactured from stainless steel, PTFE or ETFE, allowing the F427 to be specified for pumping a wide variety of fluids. The F427 also features a low-component count enabling it to be stripped down for regular cleaning or sterilization to avoid cross-contamination of products. The entire outer tube can be removed by unscrewing a single nut, giving unhindered access for cleaning. Wear-resistant seals and bearings and quick action pump/motor couplings also help to ensure maximum uptime.

Flux also offers a comprehensive range of pump motors and accessories compliant with its sanitary pumps. Flux offers its customers the reassurance of comprehensive technical support and a same- or next-day spares delivery service.

*For more information contact: Brian Wigley, Flux Pumps International (UK) Ltd., 12 Enterprise Park, Blackmoor Road, Verwood, Dorset BH31 6YS, UK. Tel: + 44 (0)1202 823304; Fax: + 44 (0)1202 813387*

### New INCA Energy hardware and EDS

New hardware by Oxford Instruments on the complete INCA Microanalysis Suite is featured on the latest version of the INCA platform and provides dramatically improved EDS accuracy, especially at low keV. The new hardware hosts SiteLock, the new beam drift correction software for the INCA Energy system for energy dispersive spectroscopy (EDS). It also hosts innovations such as the INCA Export and INCA Viewer systems that enable images, maps and spectra to be sent for manipulation on PCs not themselves running INCA. Compatible with a Windows 2000 operating system, the new hardware uses the latest industry standards such as 1394 connectivity, is CE marked and FCC Class A tested.

The INCA Microanalysis Suite comprises INCA Energy for EDS, INCA Wave for wavelengh-dispersive spectrometry (WDS) and INCA Crystal for electron backscatter diffraction (EBSD). All three techniques are available singly or in combination on a PC-based system. The system additionally offers modularity that will enable pioneering new processors to be added over the next decade without re-engineering.

*For more information contact: Oxford Instruments Analytical, Halifax Road, High Wycombe HP12 3SE, UK. Tel: + 44 (0) 1494 442255; Fax: + 44 (0)1494 524129; e-mail: analytical @oxinst.co.uk; URL http://www.oxford-instruments.com*

